# Epidemiology of open and closed diastemata in equine cheek teeth and associations with periodontal pathology

**DOI:** 10.1007/s11259-026-11216-1

**Published:** 2026-04-23

**Authors:** Fernando Mata, Claire Johnson

**Affiliations:** 1 Estação Zootécnica Nacional, Instituto Nacional de Investigação Agrária e Veterinária, Quinta da Fonte Boa, Vale de Santarém, 2005-424 Portugal; 2 Centre for Research and Development in Agrifood Systems and Sustainability, Instituto Politécnico de Viana do Castelo, Rua da Escola Industrial e Comercial Nun’Alvares 34, Viana do Castelo, 4900-347 Portugal; 3 Independent Researcher, Gloucester, United Kingdom

**Keywords:** Dentistry, Dental disease, Horse, Gum disease

## Abstract

Cheek-tooth diastemata are a common dental abnormality in horses and are considered a major predisposing factor for periodontal disease. However, the influence of diastema type and anatomical location on disease severity remains incompletely understood. To determine the prevalence and distribution of open and closed cheek-tooth diastemata and evaluate their association with periodontal disease severity in horses, while assessing the influence of age, sex, jaw location and laterality, fifty equine cadaver heads obtained from an abattoir were examined. Diastemata were identified through visual and manual examination and classified as open or closed. Periodontal disease severity was graded using a previously described clinical scoring system (0–4). Statistical analysis included multinomial logistic regression modelling to assess associations between scores of periodontal disease and explanatory variables ‘Jaw’ (‘mandible’, ‘maxilla’) and ‘Diastema’ (‘open’, ‘closed’), and ‘Age of the horse’. Non-parametric testing was also used for comparisons in periodontal disease severity. A total of 317 diastemata were identified, of which 63.1% were open and 36.9% closed. Diastemata were more frequently observed in the maxilla (68.8%) than the mandible (31.2%). Periodontal disease was present in 62.5% of diastemata. Open diastemata were significantly associated with higher periodontal disease scores. Mandibular sites showed greater disease severity despite a lower overall prevalence of diastemata. Age was associated with increased periodontal disease severity, with advanced lesions more common in older horses, particularly in mandibular arcades containing open diastemata. Mares demonstrated higher odds of open diastemata and greater periodontal disease severity compared with geldings. In conclusion, cheek-tooth diastemata, particularly open diastemata and mandibular lesions, are strongly associated with periodontal disease in horses. Early detection and targeted dental examination are essential to improve preventative management and reduce disease progression.

## Introduction

Equine cheek-tooth diastemata are defined as abnormal interdental spaces between adjacent teeth within the same dental arcade and are widely recognised as a clinically significant dental disorder in horses (Carmalt 2003). These gaps break normal tooth contact, trap food, and promote gum inflammation and periodontal damage. Prevalence varies due to population and diagnostic differences. (Carmalt 2003; Ramzan and Palmer 2011).

Cheek-tooth diastemata are considered substantially under-diagnosed in live horses, as small or functionally closed interdental spaces may be difficult to detect without sedation, full-mouth speculum examination and appropriate lighting (Tinsley et al. 2023). Therefore, post-mortem studies and detailed clinical surveys remain essential for understanding its epidemiology and pathological impact. Diastemata are congenital or acquired; congenital forms arise from abnormal dental development. (Nuttall and Ravenhill 2019; Liuti et al. 2022; Tinsley et al. 2023). Acquired diastemata develop secondary to a range of processes, including dental displacement, tooth loss, crown fracture, malocclusion, and iatrogenic factors such as premature removal of deciduous teeth (Liuti et al. 2022; Tinsley et al. 2023). Age-related changes in cheek-tooth morphology also contribute to diastema formation, as progressive occlusal wear and reduction of reserve crown height diminish rostro-caudal compression within the dental arcades (Dixon et al. 2022).

From a functional perspective, diastemata are commonly categorised as open or closed (valve) based on their occlusal geometry and feed flow dynamics (Carmalt 2003). Open diastemata allow feed entry and exit, while closed forms trap material. Closed types cause painful impaction, yet open diastemata also significantly contribute to periodontal disease (Dixon et al. 2022).

Equine periodontal disease develops when feed trapped in diastemata decomposes, causing gingival inflammation, pocket formation, and progressive ligament and bone destruction. (Occhiogrosso et al. 2023). Periodontal disease in horses is recognised as a slowly progressive condition, often affecting older animals and frequently remaining subclinical until advanced scores are reached (Occhiogrosso et al. 2023).

Recent large studies confirm horses with diastemata have significantly higher risk of periodontal disease than unaffected horses (Nuttall and Ravenhill 2019). Studies show both conditions mainly affect mandibular cheek teeth, increase with age, and vary by type, location, and severity, indicating multifactorial causes. Beyond anatomical and age factors, sex may influence periodontal disease risk. Sexual dimorphism in equine cheek teeth exists, and hormonal and immunological differences may affect susceptibility and progression (Ramzan et al. 2009). Recent studies demonstrate sex-specific differences in immune responses, inflammatory regulation and oral microbiome composition, all of which may contribute to variation in periodontal disease expression (Del Pinto et al. 2024). However, the relevance of these mechanisms in equine periodontal disease remains largely unexplored.

Although diastemata are clinically important, data on open versus closed prevalence, distribution, and links to disease severity remain limited. This study examined cadaver heads to assess prevalence, classification, and associations with periodontal disease, jaw location, age, and sex.

## Materials and methods

### Specimens and sampling strategy

A total of 50 equine cadaver heads were examined in this study. All specimens were obtained from a commercial abattoir (Potters’ Abattoir, Taunton, Somerset, United Kingdom). Sampling was opportunistic, with inclusion limited to horses presented for slaughter on a single collection day. No selection criteria were applied beyond specimen availability, and all heads were included irrespective of age, sex, or dental status.

### Oral examination

Each cadaver head received a detailed post-mortem oral examination. Periodontal disease severity was scored using Klugh’s (2005) equine grading system, ranging from 0 (no disease) to 4 (severe pathology), based on gingival inflammation, pocketing, feed impaction, and tissue destruction. Scores were recorded for statistical analysis.

Cheek-tooth diastemata were identified via visual inspection with a dental mirror and light, systematically examining all interdental spaces. Diastemata were classified as open, allowing feed ingress and egress, or closed, narrowing occlusion and restricting feed clearance, following Carmalt’s (2003) functional definitions.

### Statistical analysis

Periodontal disease scores were treated as the dependent variable in a multinomial logistic regression model, with sex (gelding, mare), jaw (maxilla, mandible), and diastema type (open, closed) as categorical predictors, and age as a continuous covariate. A full factorial model was fitted using the CSLOGISTIC routine, followed by backward stepwise elimination to retain variables significantly predicting disease scores. Model fit was assessed via pseudo R² and likelihood ratio chi-square tests, with individual predictors evaluated using Wald χ² tests. Non-parametric analyses included Mann–Whitney U tests for sex, jaw, and diastema, Student’s t-tests for age, Spearman’s correlation for age versus disease severity, and Chi-squared goodness-of-fit test to compare distribution of diastema across the dentition. Analyses used IBM SPSS Statistics 29.0.2.0 (20), with significance set at *p* < 0.05.

## Results

**Fig. 1:**
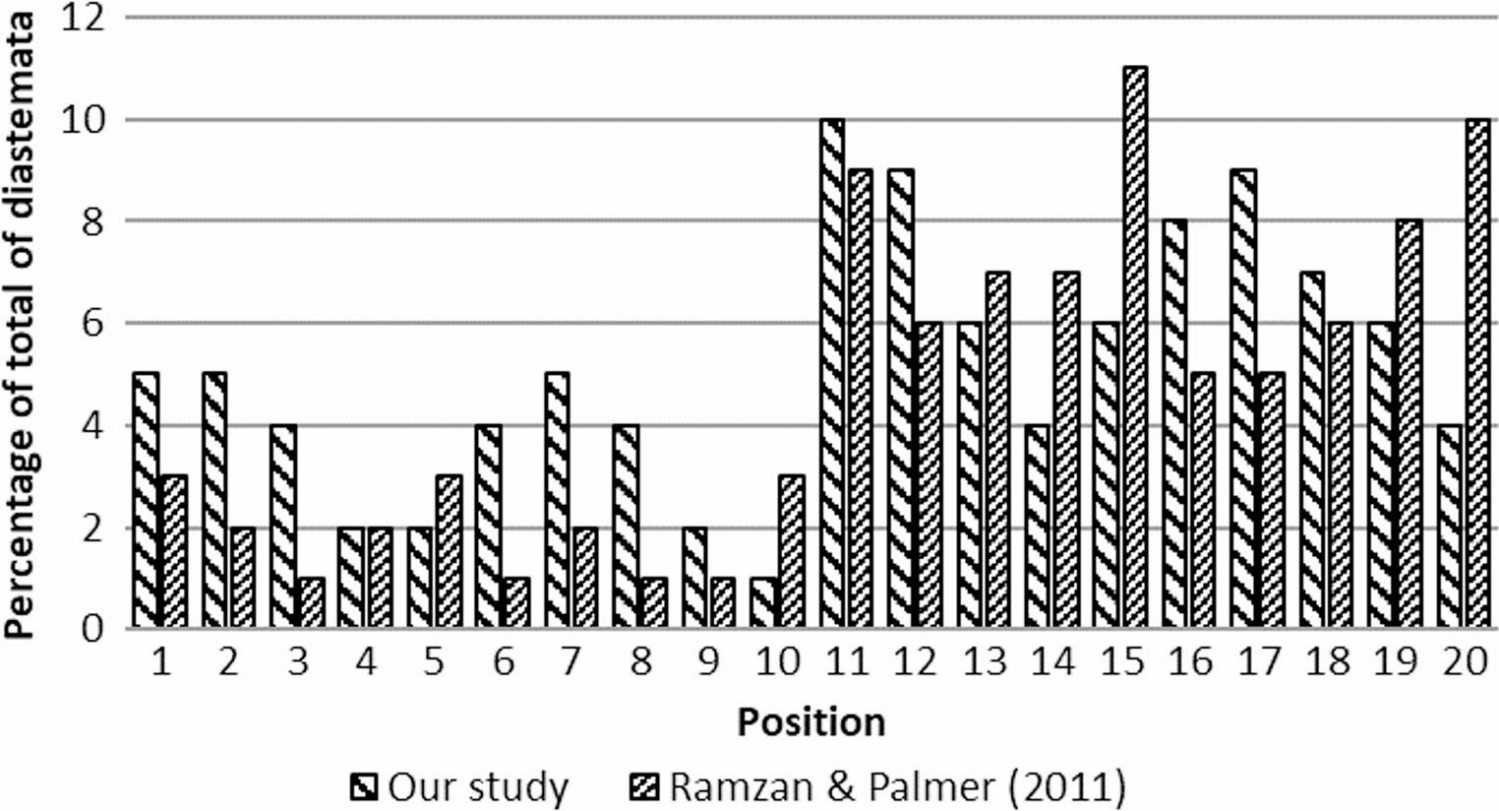
Percentage distribution of diastemata across interproximal sites of equine cheek teeth. Positions are arranged from the midline outward as follows: right maxilla (1–5), left maxilla (6–10), left mandible (11–15), and right mandible (16–20)

In 50 equine cadaver heads, 317 cheek-tooth diastemata were identified: 63.1% open and 36.9% closed. Most occurred in maxillary arcades (68.8%), with 62.5% associated with periodontal disease. Chi-square analysis showed significant distribution differences (χ² = 293.7, *P* < 0.001), compared to the previous report from Ramzan and Palmer (2011) (Fig. 1).

The score of periodontal disease differed significantly between several groups. Mares exhibited higher periodontal disease score (median = 2) than geldings (median = 1) (Mann–Whitney U = 7261, *P* < 0.01). Periodontal disease severity was also greater in association with open diastemata (median = 2) compared with closed diastemata (median = 0) (Mann–Whitney U = 7568, *P* < 0.001). In addition, mandibular diastemata were linked to higher periodontal disease score (median = 2) than those in the maxilla (median = 1) (Mann–Whitney U = 8540, *P* < 0.001). Age did not differ significantly between horses with open and closed diastemata (Student’s *t* = 1.142, *P* > 0.05), and no significant correlation was detected between age and periodontal disease score (Spearman’s *ρ* = 0.051, *P* > 0.05).

The multinomial logistic regression model was successfully fitted to the data (likelihood ratio chi-square = 495.14, 16 df, *p* < 0.001). Full parameters of the model are presented in Table 1.

**Table 1:** Multinomial logistic regression equation parameters to model “Score of Periodontal Disease”, as a function of the factors ‘Diastema’ and ‘Jaw’, and the covariate ‘Age’

Periodontal Dis score	Variable	β	SE	Wald χ^2^	df	P-value	e^*β*^	e^*β*^ 95% CI	
								lower	upper
0	Intercept	3.432	1.096	9.805	1	0.002			
	Jaw maxilla	4.015	2.022	3.943	1	0.047	55.42	1.05	2915.90
	mandible	reference							
	Diastema open	-3.462	1.052	10.830	1	< 0.001	0.03	0.01	0.25
	closed	reference							
	Age (years)	0.030	0.040	0.550	1	0.458	1.03	0.95	1.12
	maxilla * Age (years)	-0.117	0.123	0.895	1	0.344	0.89	0.70	1.13
	mandible * Age (years)	reference							
1	Intercept	2.631	1.152	5.213	1	0.022			
	Jaw maxilla	2.939	2.440	1.451	1	0.228	18.89	0.16	2254.99
	mandible	reference							
	Diastema open	-2.663	1.111	5.745	1	0.017	0.07	0.01	0.62
	closed	reference							
	Age (years)	-0.034	0.049	0.478	1	0.489	0.97	0.88	1.07
	maxilla * Age (years)	-0.255	0.207	1.522	1	0.217	0.78	0.52	1.16
	mandible * Age (years)	reference							
2	Intercept	3.258	1.096	8.833	1	0.003			
	Jaw maxilla	1.634	2.049	0.636	1	0.43	5.13	0.09	284.6
	madible	reference							
	Diastema open	-2.907	1.053	7.629	1	0.01	0.06	0.01	0.43
	closed	reference							
	Age (years)	0.018	0.040	0.204	1	0.65	1.02	0.94	1.10
	maxilla * Age (years)	0.00007	0.124	0.00001	1	1.00	1.00	0.78	1.28
	mandible * Age (years)	reference							
3	Intercept	1.972	1.149	2.943	1	0.086			
	Jaw maxilla	0.923	2.090	0.195	1	0.659	2.52	0.04	151.25
	mandible	reference							
	Diastema open	-1.088	1.105	0.968	1	0.325	0.337	0.04	2.94
	closed	reference							
	Age (years)	-0.016	0.041	0.146	1	0.702	0.98	0.91	1.07
	maxilla * Age (years)	0.030	0.127	0.056	1	0.813	1.03	0.804	1.321
	mandible * Age (years)	reference							

*Dis* disease, *SE* standard error, *CI* confidence interval, *df* degrees of freedom

**Fig. 2:**
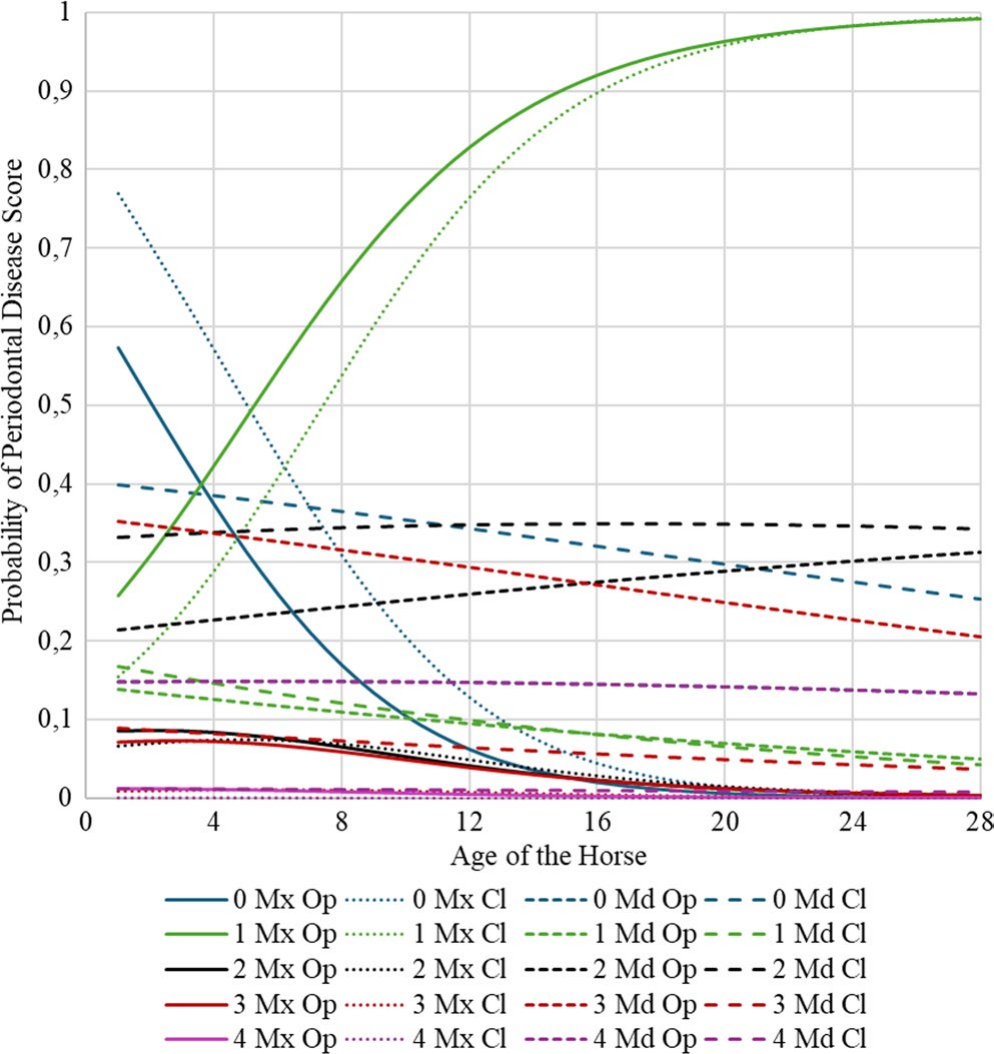
**‘**Scores of Periodontal Disease’ function of the ‘Age of the horse’, ‘Jaw’ (‘mandible’ vs ‘maxilla’), and ‘Diastema’ (‘open’ vs closed’). To note that the probabilities of the different combinations of ‘Jaw’ and ‘Diastema’ equal one. Legend: 0,1,2,3,4 ‘Score of periodontal disease’, Mx ‘maxilla’, Md ‘mandible’, Op ‘open diastema’, Cl ‘Closed diastema’

In Fig. 2, we can observe the periodontal disease scores across diastema types (open, closed), jaws (maxilla, mandible), and horse age. The probability of score 1 rises with age in the maxillae, while score 2 increases in the mandibles. Despite no correlation between age and degree of periodontal disease, within different degrees of periodontal disease, we can observe age- and place- (maxilla, mandible) related differences: lower scores dominate in younger horses, higher scores (2–4) increase with age, particularly in mandibles with open diastemata. Overall, zero scores decline with age, indicating higher periodontal disease prevalence in older horses.

## Discussion

Nuttall and Ravenhill (2019) reported higher diastema prevalence in mandibular cheek teeth, a pattern contradicted by our results, with the maxilla containing more interdental spaces overall than the mandibles. Considering diastema type, maxillary diastemata were significantly more often open (*P* < 0.05), highlighting the need to assess both anatomical location and functional morphology. No consistent positional pattern was found for diastemata across individual cheek-tooth sites, aside from higher maxillary frequency. Significant differences from Ramzan and Palmer (2011) (χ² = 293.7, *P* < 0.001) suggest influences of age, breed, occlusal wear, and methodology.

A strong link exists between diastemata and periodontal disease, with 62.5% showing pathology, aligning with prior reports of interdental feed causing inflammation. (Simhofer et al. 2008). Recent studies confirm horses with diastemata, especially older ones, are significantly more likely to develop periodontal disease than those without gaps (Nuttall and Ravenhill 2019; Dixon et al. 2021).

Although open diastemata have traditionally been considered less clinically significant because of their reduced propensity for food impaction, this study found a significant association between open diastemata and increased periodontal disease severity (*P* < 0.001). Scores were higher with open versus closed diastemata (*P* < 0.01), indicating open diastemata contribute to chronic periodontal breakdown through feed retention, bacterial growth, and inflammation. These results support the concept of periodontal disease as a progressive condition driven by prolonged exposure to impacted feed, rather than acute obstruction alone, consistent with recent studies (Vlaminck et al. 2006; Dixon et al. 2021).

Age has been widely reported as a major risk factor for both diastemata and periodontal disease (Carmalt 2003; Du Toit et al. 2009; Kennedy and Dixon 2018). However, no significant associations were directly identified in the present study between age, diastema type or periodontal disease severity (*P* > 0.05). The presence of interactions between the factors considered in our study and the covariate “age” must be considered. However, age-related variation in periodontal disease, while considering the factors analysed, supports evidence that equine periodontal pathology progresses over life.

Sex-related differences were identified, with mares exhibiting both a higher degree of periodontal disease and increased odds of having open rather than closed diastemata (*P* < 0.01). This observation has not been widely documented in the equine dental literature, although Ramzan et al. (2009) previously noted sexual dimorphism in equine cheek-tooth morphology. Comparable sex-associated differences in oral health have been reported in humans, where higher salivary calcium concentrations in males have been correlated with improved dental health (Sewón et al. 1998). More recent human and veterinary studies suggest that hormonal and immunological differences between sexes may influence periodontal disease susceptibility and progression, warranting further investigation in equine populations (Del Pinto et al. 2024).

Younger horses show lower scores due to intact interdental contact, whereas age-related occlusal wear and crown height reduction increase diastema formation, feed impaction, bacterial colonisation, and tissue destruction. Older horses exhibit higher disease prevalence and severity (Occhiogrosso et al. 2023). The higher likelihood of moderate periodontal disease (score 2) in the mandible may reflect anatomical predispositions promoting feed retention and deeper pockets, with age-related histological changes, including inflammation and attachment loss, often linked to interdental spacing abnormalities (Zapf et al. 2023). The higher prevalence of severe periodontal lesions (scores 3–4) in older horses, especially in mandibular open diastemata, reflects chronic feed accumulation, microbial proliferation, and progressive ligament and bone destruction, highlighting the importance of early detection and regular dental monitoring (Nuttall and Ravenhill et al. 2019). These findings emphasize age, diastema type, and jaw location jointly influence periodontal disease severity, underscoring the need for targeted dental care in older horses.

Despite providing valuable insights, this study has limitations. Using cadaver heads from a convenience sample may introduce selection bias and limit generalisability. Its cross-sectional design prevents conclusions about causality between diastema type and periodontal disease. Visual and manual assessments may underestimate subtle or early lesions. Future research should involve larger, prospectively sampled live populations and incorporate advanced imaging (radiography, CT, endoscopy) to characterise diastemata and early periodontal changes. Longitudinal studies are needed to track diastema development, its impact on disease progression, and interactions with breed, sex, diet, and management, improving risk assessment and preventative strategies.

## Data Availability

Data will be made available from the corresponding author under a reseonable request.
